# Crystal structure of cyproconazole

**DOI:** 10.1107/S2056989015022665

**Published:** 2015-11-28

**Authors:** Gihaeng Kang, Jineun Kim, Eunjin Kwon, Tae Ho Kim

**Affiliations:** a Department of Chemistry and Research Institute of Natural Sciences, Gyeongsang National University, Jinju 52828, Republic of Korea

**Keywords:** crystal structure, cyproconazole, butan-2-ol, fungicidal properties, hydrogen bonding

## Abstract

The title compound [systematic name: 2-(4-chloro­phen­yl)-3-cyclo­propyl-1-(1*H*-1,2,4-triazol-1-yl)butan-2-ol], C_15_H_18_ClN_3_O, is a conazole fungicide. The asymmetric unit comprises two enanti­omeric pairs (mol­ecules *A* and *B*) in which the dihedral angles between the chloro­phenyl and triazole rings are 46.54 (9) (mol­ecule *A*) and 67.03 (8)° (mol­ecule *B*). In the crystal, C—H⋯O, O—H⋯N and C—H⋯Cl hydrogen bonds and weak C—H⋯π inter­actions [3.473 (2) Å] link adjacent mol­ecules, forming columns along the *a* axis.

## Related literature   

For information on the fungicidal properties of the title compound, see: Hester *et al.* (2012 ▸). For a related crystal structure, see: Chopra *et al.* (2004 ▸).
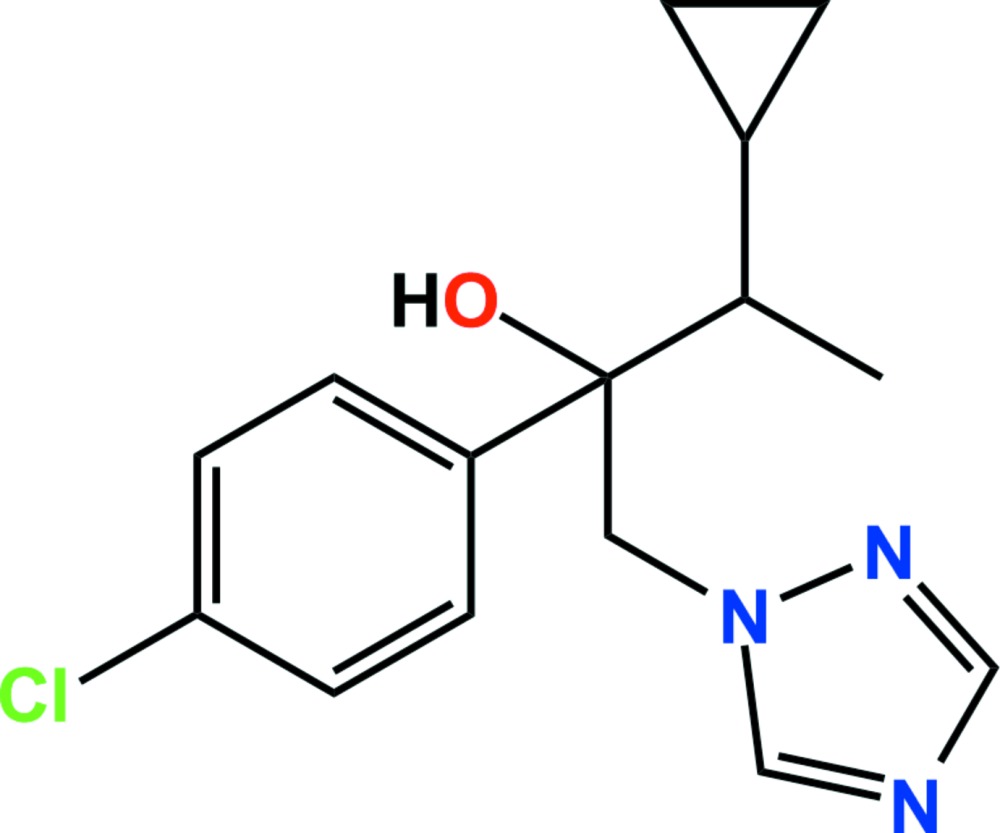


## Experimental   

### Crystal data  

C_15_H_18_ClN_3_O
*M* *_r_* = 291.77
Triclinic, 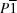

*a* = 9.7783 (11) Å
*b* = 12.2150 (13) Å
*c* = 14.5861 (15) Å
α = 107.965 (6)°
β = 108.254 (5)°
γ = 99.533 (5)°
*V* = 1506.1 (3) Å^3^
*Z* = 4
Mo *K*α radiation
μ = 0.25 mm^−1^
*T* = 173 K
0.27 × 0.24 × 0.13 mm

### Data collection  

Bruker APEXII CCD diffractometer
Absorption correction: multi-scan (*SADABS*; Bruker, 2013 ▸) *T*_min_ = 0.657, *T*_max_ = 0.746
24278 measured reflections
5902 independent reflections
4637 reflections with *I* > 2σ(*I*)
*R*_int_ = 0.047

### Refinement  

*R*[*F* ^2^ > 2σ(*F* ^2^)] = 0.048
*wR*(*F* ^2^) = 0.142
*S* = 1.10
5902 reflections
365 parameters
H-atom parameters constrained
Δρ_max_ = 0.37 e Å^−3^
Δρ_min_ = −0.42 e Å^−3^

### 

Data collection: *APEX2* (Bruker, 2013 ▸); cell refinement: *SAINT* (Bruker, 2013 ▸); data reduction: *SAINT*; program(s) used to solve structure: *SHELXS97* (Sheldrick, 2008 ▸); program(s) used to refine structure: *SHELXL2013* (Sheldrick, 2015 ▸); molecular graphics: *DIAMOND* (Brandenburg, 2010 ▸); software used to prepare material for publication: *SHELXTL* (Sheldrick, 2008 ▸).

## Supplementary Material

Crystal structure: contains datablock(s) global, I. DOI: 10.1107/S2056989015022665/hg5465sup1.cif

Structure factors: contains datablock(s) I. DOI: 10.1107/S2056989015022665/hg5465Isup2.hkl

Supporting information file. DOI: 10.1107/S2056989015022665/hg5465Isup3.cml

**Fig. 1.**
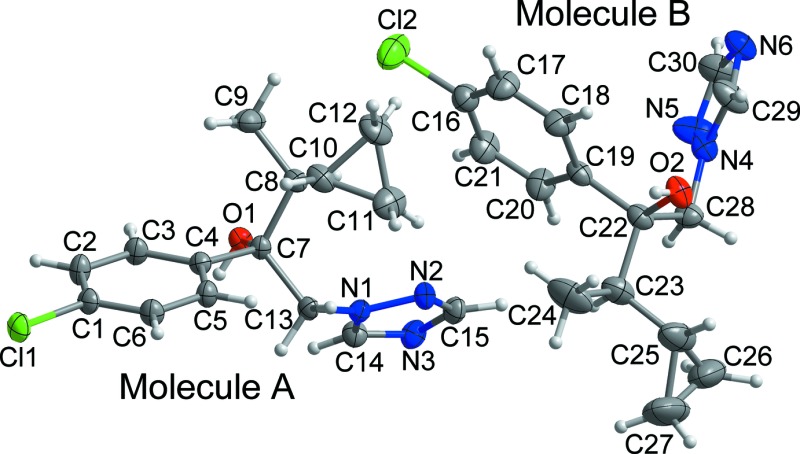
The asymmetric unit of the title compound with the atom-numbering scheme. Displacement ellipsoids are drawn at the 50% probability level. H atoms are shown as small spheres of arbitrary radius.

**Fig. 2.**
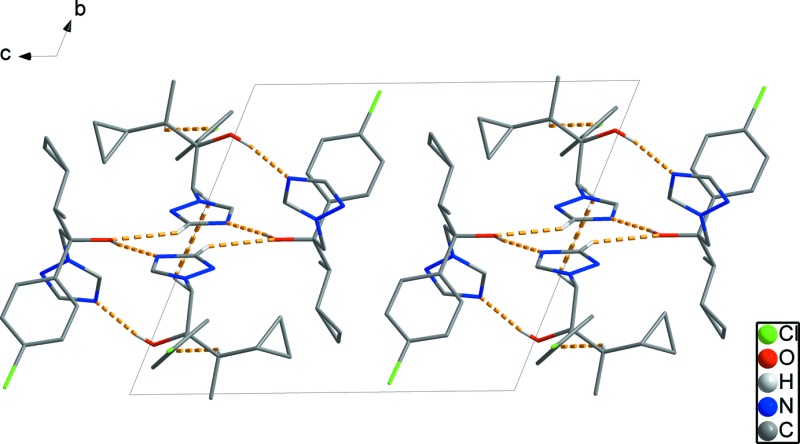
Crystal packing viewed along the a axis. The intermolecular interactions are shown as dashed lines.

. DOI: 10.1107/S2056989015022665/hg5465fig1.tif
The asymmetric unit of the title compound with the atom-numbering scheme. Displacement ellipsoids are drawn at the 50% probability level. H atoms are shown as small spheres of arbitrary radius.

a . DOI: 10.1107/S2056989015022665/hg5465fig2.tif
Crystal packing viewed along the *a* axis. The inter­molecular inter­actions are shown as dashed lines.

CCDC reference: 1439054

Additional supporting information:  crystallographic information; 3D view; checkCIF report

## Figures and Tables

**Table 1:** Hydrogen-bond geometry (Å, °) *Cg*1 is the centroid of the N1/N2/C15/N3/C14 ring.

*D*—H⋯*A*	*D*—H	H⋯*A*	*D*⋯*A*	*D*—H⋯*A*
O1—H1⋯N6^i^	0.84	2.05	2.884 (2)	171
O2—H2⋯N3^ii^	0.84	2.07	2.856 (2)	156
C8—H8⋯Cl1^iii^	1.00	2.83	3.633 (2)	137
C15—H15⋯O2^iv^	0.95	2.53	3.284 (2)	137
C13—H13*B*⋯*Cg*1^v^	1.00	2.91	3.473 (2)	117

Symmetry codes: (i) 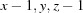; (ii) 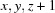; (iii) 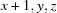; (iv) 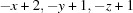; (v) 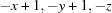.

## supplementary crystallographic information

### S1. Comment

Cyproconazole [systematic name: 2-(4-chlorophenyl)-3-cyclopropyl-1-(1*H*-1,2,4-triazol-1-yl)butan-2-ol] is a conazole fungicide used as agricultural pesticides and pharmaceutical products. (Hester *et al.*, 2012). Its crystal structure is reported herein. In this compound (Fig. 1), there are two enantiomeric pairs (Molecule A and B) in the asymmetric unit, with the dihedral angles between the chlorophenyl and triazole rings are 46.54 (9) (Molecule A) and 67.03 (8)° (Molecule B), respectively. All bond lengths and bond angles are normal and comparable to those observed in the crystal structure of a similar compound (Chopra *et al.*, 2004).

In the crystal structure (Fig. 2), the crystal structure is stabilized by C—H···O, O—H···N, and C—H···Cl hydrogen bonds (Table 1), as well as intermolecular C13—H13B···*Cg*1^v^(*Cg*1 is the centroid of the N1—N2—C15—N3—C14 ring) interaction with a triazole ring are present, resulting in one-dimensional columns along to *a*-axis [for symmetry code: (v), -*x* + 1, -*y* + 1, -*z*].

### S2. Experimental

The title compound was purchased from the Dr. Ehrenstorfer GmbH Company. Slow evaporation of a solution in CH_2_Cl_2_ gave single crystals suitable for X-ray analysis.

### S3. Refinement

All H-atoms were positioned geometrically and refined using a riding model with d(O—H) = 0.84 Å, *U*_iso_ = 1.5*U*_eq_(C) for O—H group, d(C—H) = 1.00 Å, *U*_iso_ = 1.2*U*_eq_(C) for C*sp*^3^—H group, d(C—H) = 0.99 Å, *U*_iso_ = 1.2*U*_eq_(C) for CH_2_ group, d(C—H) = 0.98 Å, *U*_iso_ = 1.5*U*_eq_(C) for CH_3_ group, d(C—H) = 0.95 Å, *U*_iso_ = 1.2*U*_eq_(C) for aromatic C—H.

### Crystal data

**Table:**

C_15_H_18_ClN_3_O	*Z* = 4
*M**_r_* = 291.77	*F*(000) = 616
Triclinic, *P*1	*D*_x_ = 1.287 Mg m^−^^3^
*a* = 9.7783 (11) Å	Mo *K*α radiation, λ = 0.71073 Å
*b* = 12.2150 (13) Å	Cell parameters from 6577 reflections
*c* = 14.5861 (15) Å	θ = 2.2–27.7°
α = 107.965 (6)°	µ = 0.25 mm^−^^1^
β = 108.254 (5)°	*T* = 173 K
γ = 99.533 (5)°	Block, colourless
*V* = 1506.1 (3) Å^3^	0.27 × 0.24 × 0.13 mm

### Data collection

**Table:**

Bruker APEXII CCD diffractometer	4637 reflections with *I* > 2σ(*I*)
φ and ω scans	*R*_int_ = 0.047
Absorption correction: multi-scan (*SADABS*; Bruker, 2013)	θ_max_ = 26.0°, θ_min_ = 1.6°
*T*_min_ = 0.657, *T*_max_ = 0.746	*h* = −12→12
24278 measured reflections	*k* = −15→15
5902 independent reflections	*l* = −17→17

### Refinement

**Table:**

Refinement on *F*^2^	0 restraints
Least-squares matrix: full	Hydrogen site location: inferred from neighbouring sites
*R*[*F*^2^ > 2σ(*F*^2^)] = 0.048	H-atom parameters constrained
*wR*(*F*^2^) = 0.142	*w* = 1/[σ^2^(*F*_o_^2^) + (0.0829*P*)^2^] where *P* = (*F*_o_^2^ + 2*F*_c_^2^)/3
*S* = 1.10	(Δ/σ)_max_ < 0.001
5902 reflections	Δρ_max_ = 0.37 e Å^−^^3^
365 parameters	Δρ_min_ = −0.42 e Å^−^^3^

### Special details

**Table:**

Geometry. All e.s.d.'s (except the e.s.d. in the dihedral angle between two l.s. planes) are estimated using the full covariance matrix. The cell e.s.d.'s are taken into account individually in the estimation of e.s.d.'s in distances, angles and torsion angles; correlations between e.s.d.'s in cell parameters are only used when they are defined by crystal symmetry. An approximate (isotropic) treatment of cell e.s.d.'s is used for estimating e.s.d.'s involving l.s. planes.

### Fractional atomic coordinates and isotropic or equivalent isotropic displacement parameters (Å^2^)

**Table:**

	*x*	*y*	*z*	*U*_iso_*/*U*_eq_	
Cl1	−0.04911 (5)	0.86025 (4)	0.05469 (4)	0.03034 (16)	
Cl2	0.80245 (8)	0.97120 (5)	0.66620 (5)	0.0565 (2)	
O1	0.60192 (13)	0.83935 (11)	0.01077 (9)	0.0229 (3)	
H1	0.5303	0.8046	−0.0480	0.034*	
O2	0.92927 (15)	0.49775 (13)	0.78653 (10)	0.0331 (3)	
H2	0.8646	0.5125	0.8114	0.050*	
N1	0.65641 (16)	0.62487 (13)	0.02174 (12)	0.0224 (3)	
N2	0.76461 (17)	0.59337 (14)	0.08493 (12)	0.0256 (4)	
N3	0.77957 (19)	0.55315 (14)	−0.07288 (13)	0.0301 (4)	
N4	1.14669 (17)	0.56158 (14)	0.69780 (12)	0.0278 (4)	
N5	1.2121 (2)	0.60435 (18)	0.64189 (14)	0.0476 (6)	
N6	1.35778 (19)	0.69573 (17)	0.81207 (13)	0.0380 (4)	
C1	0.1300 (2)	0.84834 (16)	0.06922 (14)	0.0243 (4)	
C2	0.2145 (2)	0.91250 (17)	0.03227 (15)	0.0281 (4)	
H2A	0.1766	0.9649	0.0014	0.034*	
C3	0.3549 (2)	0.89902 (17)	0.04108 (15)	0.0273 (4)	
H3	0.4130	0.9431	0.0157	0.033*	
C4	0.41388 (19)	0.82362 (16)	0.08560 (14)	0.0208 (4)	
C5	0.3266 (2)	0.76251 (16)	0.12487 (14)	0.0244 (4)	
H5	0.3652	0.7117	0.1576	0.029*	
C6	0.1865 (2)	0.77470 (17)	0.11700 (15)	0.0259 (4)	
H6	0.1290	0.7329	0.1442	0.031*	
C7	0.56362 (19)	0.80332 (16)	0.08570 (13)	0.0198 (4)	
C8	0.6976 (2)	0.87845 (16)	0.19188 (14)	0.0233 (4)	
H8	0.7890	0.8564	0.1850	0.028*	
C9	0.7281 (3)	1.01291 (18)	0.21553 (17)	0.0390 (5)	
H9A	0.6417	1.0384	0.2248	0.059*	
H9B	0.7438	1.0291	0.1573	0.059*	
H9C	0.8183	1.0575	0.2797	0.059*	
C10	0.6798 (2)	0.85291 (18)	0.28317 (15)	0.0311 (5)	
H10	0.5898	0.8689	0.2972	0.037*	
C11	0.7297 (3)	0.7547 (2)	0.31196 (17)	0.0458 (6)	
H11A	0.6697	0.7110	0.3401	0.055*	
H11B	0.7740	0.7052	0.2683	0.055*	
C12	0.8179 (3)	0.8827 (2)	0.37830 (17)	0.0440 (6)	
H12A	0.9166	0.9123	0.3757	0.053*	
H12B	0.8123	0.9182	0.4474	0.053*	
C13	0.5409 (2)	0.66645 (16)	0.05308 (14)	0.0221 (4)	
H13A	0.5377	0.6444	0.1124	0.027*	
H13B	0.4421	0.6236	−0.0058	0.027*	
C14	0.6682 (2)	0.59944 (17)	−0.07065 (15)	0.0283 (4)	
H14	0.6042	0.6131	−0.1276	0.034*	
C15	0.8348 (2)	0.55172 (16)	0.02459 (15)	0.0272 (4)	
H15	0.9183	0.5226	0.0474	0.033*	
C16	0.8286 (2)	0.83582 (19)	0.67320 (16)	0.0361 (5)	
C17	0.8550 (3)	0.8183 (2)	0.76483 (17)	0.0423 (6)	
H17	0.8588	0.8798	0.8251	0.051*	
C18	0.8759 (2)	0.71082 (19)	0.76897 (15)	0.0364 (5)	
H18	0.8973	0.7001	0.8333	0.044*	
C19	0.8663 (2)	0.61851 (17)	0.68184 (14)	0.0267 (4)	
C20	0.8401 (3)	0.6393 (2)	0.58996 (16)	0.0372 (5)	
H20	0.8347	0.5777	0.5292	0.045*	
C21	0.8219 (3)	0.7468 (2)	0.58518 (16)	0.0412 (6)	
H21	0.8048	0.7596	0.5220	0.049*	
C22	0.8813 (2)	0.49691 (18)	0.68326 (14)	0.0283 (5)	
C23	0.7293 (2)	0.3970 (2)	0.60963 (17)	0.0390 (5)	
H23	0.7046	0.3983	0.5381	0.047*	
C24	0.6022 (3)	0.4221 (3)	0.6419 (2)	0.0642 (8)	
H24A	0.5083	0.3594	0.5926	0.096*	
H24B	0.5923	0.5006	0.6418	0.096*	
H24C	0.6232	0.4226	0.7123	0.096*	
C25	0.7417 (3)	0.2731 (2)	0.6014 (2)	0.0571 (8)	
H25	0.7784	0.2627	0.6695	0.069*	
C26	0.7812 (4)	0.1962 (2)	0.5174 (2)	0.0732 (10)	
H26A	0.8009	0.2299	0.4676	0.088*	
H26B	0.8432	0.1436	0.5351	0.088*	
C27	0.6240 (4)	0.1651 (3)	0.5110 (3)	0.0892 (12)	
H27A	0.5887	0.0936	0.5250	0.107*	
H27B	0.5463	0.1799	0.4574	0.107*	
C28	1.0042 (2)	0.46528 (18)	0.64609 (15)	0.0312 (5)	
H28A	1.0226	0.3928	0.6584	0.037*	
H28B	0.9680	0.4451	0.5698	0.037*	
C29	1.2336 (2)	0.6183 (2)	0.79826 (17)	0.0452 (6)	
H29	1.2103	0.6053	0.8533	0.054*	
C30	1.3371 (3)	0.6832 (2)	0.71451 (17)	0.0427 (6)	
H30	1.4084	0.7287	0.6985	0.051*	

### Atomic displacement parameters (Å^2^)

**Table:**

	*U*^11^	*U*^22^	*U*^33^	*U*^12^	*U*^13^	*U*^23^
Cl1	0.0238 (3)	0.0358 (3)	0.0387 (3)	0.0144 (2)	0.0170 (2)	0.0158 (2)
Cl2	0.0824 (5)	0.0425 (4)	0.0454 (4)	0.0272 (3)	0.0187 (3)	0.0196 (3)
O1	0.0216 (7)	0.0292 (7)	0.0215 (6)	0.0068 (5)	0.0103 (5)	0.0126 (5)
O2	0.0312 (8)	0.0497 (9)	0.0256 (7)	0.0156 (7)	0.0137 (6)	0.0189 (6)
N1	0.0230 (8)	0.0218 (8)	0.0242 (8)	0.0085 (6)	0.0101 (7)	0.0091 (6)
N2	0.0243 (8)	0.0296 (9)	0.0269 (9)	0.0123 (7)	0.0103 (7)	0.0133 (7)
N3	0.0362 (10)	0.0304 (9)	0.0309 (9)	0.0152 (7)	0.0184 (8)	0.0127 (7)
N4	0.0270 (9)	0.0344 (9)	0.0236 (8)	0.0104 (7)	0.0116 (7)	0.0108 (7)
N5	0.0502 (12)	0.0563 (13)	0.0248 (9)	−0.0060 (10)	0.0203 (9)	0.0062 (8)
N6	0.0299 (10)	0.0504 (11)	0.0298 (10)	0.0053 (8)	0.0076 (8)	0.0180 (8)
C1	0.0211 (9)	0.0265 (10)	0.0283 (10)	0.0104 (7)	0.0132 (8)	0.0089 (8)
C2	0.0303 (11)	0.0330 (11)	0.0340 (11)	0.0166 (9)	0.0169 (9)	0.0211 (9)
C3	0.0250 (10)	0.0298 (10)	0.0356 (11)	0.0096 (8)	0.0165 (9)	0.0180 (9)
C4	0.0206 (9)	0.0215 (9)	0.0206 (9)	0.0061 (7)	0.0091 (7)	0.0071 (7)
C5	0.0256 (10)	0.0270 (10)	0.0266 (10)	0.0113 (8)	0.0122 (8)	0.0142 (8)
C6	0.0244 (10)	0.0303 (10)	0.0294 (10)	0.0086 (8)	0.0143 (8)	0.0153 (8)
C7	0.0194 (9)	0.0227 (9)	0.0222 (9)	0.0078 (7)	0.0104 (7)	0.0119 (7)
C8	0.0222 (9)	0.0261 (10)	0.0207 (9)	0.0066 (7)	0.0080 (8)	0.0085 (7)
C9	0.0430 (13)	0.0281 (11)	0.0307 (11)	0.0020 (9)	0.0041 (10)	0.0062 (9)
C10	0.0278 (10)	0.0427 (12)	0.0238 (10)	0.0084 (9)	0.0115 (9)	0.0134 (9)
C11	0.0645 (16)	0.0458 (14)	0.0286 (11)	0.0150 (12)	0.0147 (11)	0.0201 (10)
C12	0.0447 (14)	0.0547 (15)	0.0246 (11)	0.0090 (11)	0.0057 (10)	0.0158 (10)
C13	0.0198 (9)	0.0236 (9)	0.0253 (9)	0.0078 (7)	0.0110 (8)	0.0093 (8)
C14	0.0310 (11)	0.0308 (11)	0.0244 (10)	0.0118 (8)	0.0112 (9)	0.0107 (8)
C15	0.0264 (10)	0.0271 (10)	0.0331 (11)	0.0119 (8)	0.0147 (9)	0.0126 (8)
C16	0.0406 (12)	0.0354 (12)	0.0302 (11)	0.0120 (9)	0.0109 (10)	0.0124 (9)
C17	0.0578 (15)	0.0382 (13)	0.0262 (11)	0.0153 (11)	0.0161 (11)	0.0059 (9)
C18	0.0492 (13)	0.0363 (12)	0.0218 (10)	0.0097 (10)	0.0135 (10)	0.0104 (9)
C19	0.0240 (10)	0.0354 (11)	0.0182 (9)	0.0078 (8)	0.0078 (8)	0.0079 (8)
C20	0.0515 (14)	0.0429 (13)	0.0230 (10)	0.0207 (10)	0.0185 (10)	0.0126 (9)
C21	0.0567 (14)	0.0473 (14)	0.0247 (11)	0.0203 (11)	0.0155 (10)	0.0182 (10)
C22	0.0270 (10)	0.0370 (11)	0.0193 (9)	0.0072 (8)	0.0087 (8)	0.0103 (8)
C23	0.0322 (12)	0.0445 (13)	0.0332 (12)	0.0010 (10)	0.0082 (10)	0.0157 (10)
C24	0.0351 (14)	0.088 (2)	0.0525 (16)	−0.0016 (13)	0.0166 (12)	0.0152 (14)
C25	0.0543 (16)	0.0477 (15)	0.0515 (16)	−0.0070 (12)	0.0008 (13)	0.0275 (13)
C26	0.085 (2)	0.0339 (14)	0.0665 (19)	0.0105 (14)	0.0025 (17)	0.0063 (13)
C27	0.089 (2)	0.0421 (17)	0.090 (2)	−0.0142 (16)	−0.0010 (19)	0.0186 (16)
C28	0.0318 (11)	0.0308 (11)	0.0260 (10)	0.0066 (9)	0.0114 (9)	0.0057 (8)
C29	0.0319 (12)	0.0713 (17)	0.0274 (11)	0.0010 (11)	0.0052 (10)	0.0261 (11)
C30	0.0395 (13)	0.0511 (14)	0.0331 (12)	0.0005 (10)	0.0166 (10)	0.0145 (10)

### Geometric parameters (Å, º)

**Table:**

Cl1—C1	1.7336 (19)	C11—C12	1.490 (3)
Cl2—C16	1.743 (2)	C11—H11A	0.9900
O1—C7	1.424 (2)	C11—H11B	0.9900
O1—H1	0.8400	C12—H12A	0.9900
O2—C22	1.427 (2)	C12—H12B	0.9900
O2—H2	0.8400	C13—H13A	0.9900
N1—C14	1.333 (2)	C13—H13B	0.9900
N1—N2	1.364 (2)	C14—H14	0.9500
N1—C13	1.454 (2)	C15—H15	0.9500
N2—C15	1.313 (2)	C16—C17	1.370 (3)
N3—C14	1.312 (3)	C16—C21	1.377 (3)
N3—C15	1.360 (2)	C17—C18	1.378 (3)
N4—C29	1.322 (3)	C17—H17	0.9500
N4—N5	1.351 (2)	C18—C19	1.379 (3)
N4—C28	1.465 (2)	C18—H18	0.9500
N5—C30	1.309 (3)	C19—C20	1.394 (3)
N6—C29	1.326 (3)	C19—C22	1.522 (3)
N6—C30	1.328 (3)	C20—C21	1.374 (3)
C1—C6	1.382 (3)	C20—H20	0.9500
C1—C2	1.383 (3)	C21—H21	0.9500
C2—C3	1.380 (3)	C22—C28	1.523 (3)
C2—H2A	0.9500	C22—C23	1.563 (3)
C3—C4	1.383 (3)	C23—C24	1.505 (3)
C3—H3	0.9500	C23—C25	1.509 (4)
C4—C5	1.404 (2)	C23—H23	1.0000
C4—C7	1.525 (2)	C24—H24A	0.9800
C5—C6	1.376 (3)	C24—H24B	0.9800
C5—H5	0.9500	C24—H24C	0.9800
C6—H6	0.9500	C25—C26	1.497 (4)
C7—C13	1.546 (3)	C25—C27	1.509 (4)
C7—C8	1.553 (2)	C25—H25	1.0000
C8—C10	1.511 (3)	C26—C27	1.487 (5)
C8—C9	1.526 (3)	C26—H26A	0.9900
C8—H8	1.0000	C26—H26B	0.9900
C9—H9A	0.9800	C27—H27A	0.9900
C9—H9B	0.9800	C27—H27B	0.9900
C9—H9C	0.9800	C28—H28A	0.9900
C10—C11	1.493 (3)	C28—H28B	0.9900
C10—C12	1.494 (3)	C29—H29	0.9500
C10—H10	1.0000	C30—H30	0.9500
			
C7—O1—H1	109.5	N3—C14—N1	111.26 (17)
C22—O2—H2	109.5	N3—C14—H14	124.4
C14—N1—N2	109.19 (16)	N1—C14—H14	124.4
C14—N1—C13	128.64 (16)	N2—C15—N3	115.29 (17)
N2—N1—C13	121.83 (15)	N2—C15—H15	122.4
C15—N2—N1	102.17 (15)	N3—C15—H15	122.4
C14—N3—C15	102.09 (16)	C17—C16—C21	120.6 (2)
C29—N4—N5	108.75 (16)	C17—C16—Cl2	120.43 (17)
C29—N4—C28	130.05 (17)	C21—C16—Cl2	118.95 (17)
N5—N4—C28	121.17 (15)	C16—C17—C18	119.6 (2)
C30—N5—N4	102.58 (17)	C16—C17—H17	120.2
C29—N6—C30	101.93 (18)	C18—C17—H17	120.2
C6—C1—C2	120.79 (18)	C17—C18—C19	121.46 (19)
C6—C1—Cl1	119.79 (14)	C17—C18—H18	119.3
C2—C1—Cl1	119.41 (15)	C19—C18—H18	119.3
C3—C2—C1	118.83 (18)	C18—C19—C20	117.53 (19)
C3—C2—H2A	120.6	C18—C19—C22	122.35 (17)
C1—C2—H2A	120.6	C20—C19—C22	120.12 (17)
C2—C3—C4	122.27 (17)	C21—C20—C19	121.63 (19)
C2—C3—H3	118.9	C21—C20—H20	119.2
C4—C3—H3	118.9	C19—C20—H20	119.2
C3—C4—C5	117.29 (17)	C20—C21—C16	119.11 (19)
C3—C4—C7	120.59 (16)	C20—C21—H21	120.4
C5—C4—C7	122.02 (16)	C16—C21—H21	120.4
C6—C5—C4	121.39 (17)	O2—C22—C19	112.11 (15)
C6—C5—H5	119.3	O2—C22—C28	104.26 (16)
C4—C5—H5	119.3	C19—C22—C28	110.58 (16)
C5—C6—C1	119.38 (17)	O2—C22—C23	111.03 (16)
C5—C6—H6	120.3	C19—C22—C23	109.95 (16)
C1—C6—H6	120.3	C28—C22—C23	108.73 (16)
O1—C7—C4	110.59 (14)	C24—C23—C25	111.2 (2)
O1—C7—C13	108.50 (14)	C24—C23—C22	111.75 (19)
C4—C7—C13	106.44 (13)	C25—C23—C22	112.09 (19)
O1—C7—C8	105.33 (13)	C24—C23—H23	107.2
C4—C7—C8	113.17 (15)	C25—C23—H23	107.2
C13—C7—C8	112.78 (15)	C22—C23—H23	107.2
C10—C8—C9	110.00 (16)	C23—C24—H24A	109.5
C10—C8—C7	113.93 (14)	C23—C24—H24B	109.5
C9—C8—C7	111.32 (15)	H24A—C24—H24B	109.5
C10—C8—H8	107.1	C23—C24—H24C	109.5
C9—C8—H8	107.1	H24A—C24—H24C	109.5
C7—C8—H8	107.1	H24B—C24—H24C	109.5
C8—C9—H9A	109.5	C26—C25—C27	59.3 (2)
C8—C9—H9B	109.5	C26—C25—C23	121.6 (2)
H9A—C9—H9B	109.5	C27—C25—C23	118.7 (2)
C8—C9—H9C	109.5	C26—C25—H25	115.2
H9A—C9—H9C	109.5	C27—C25—H25	115.2
H9B—C9—H9C	109.5	C23—C25—H25	115.2
C11—C10—C12	59.86 (15)	C27—C26—C25	60.7 (2)
C11—C10—C8	121.87 (19)	C27—C26—H26A	117.7
C12—C10—C8	118.46 (17)	C25—C26—H26A	117.7
C11—C10—H10	115.1	C27—C26—H26B	117.7
C12—C10—H10	115.1	C25—C26—H26B	117.7
C8—C10—H10	115.1	H26A—C26—H26B	114.8
C12—C11—C10	60.11 (14)	C26—C27—C25	59.95 (19)
C12—C11—H11A	117.8	C26—C27—H27A	117.8
C10—C11—H11A	117.8	C25—C27—H27A	117.8
C12—C11—H11B	117.8	C26—C27—H27B	117.8
C10—C11—H11B	117.8	C25—C27—H27B	117.8
H11A—C11—H11B	114.9	H27A—C27—H27B	114.9
C11—C12—C10	60.03 (14)	N4—C28—C22	113.94 (15)
C11—C12—H12A	117.8	N4—C28—H28A	108.8
C10—C12—H12A	117.8	C22—C28—H28A	108.8
C11—C12—H12B	117.8	N4—C28—H28B	108.8
C10—C12—H12B	117.8	C22—C28—H28B	108.8
H12A—C12—H12B	114.9	H28A—C28—H28B	107.7
N1—C13—C7	114.60 (14)	N4—C29—N6	111.02 (19)
N1—C13—H13A	108.6	N4—C29—H29	124.5
C7—C13—H13A	108.6	N6—C29—H29	124.5
N1—C13—H13B	108.6	N5—C30—N6	115.70 (19)
C7—C13—H13B	108.6	N5—C30—H30	122.2
H13A—C13—H13B	107.6	N6—C30—H30	122.2
			
C14—N1—N2—C15	0.54 (18)	N1—N2—C15—N3	−0.4 (2)
C13—N1—N2—C15	174.41 (15)	C14—N3—C15—N2	0.2 (2)
C29—N4—N5—C30	1.2 (3)	C21—C16—C17—C18	0.4 (4)
C28—N4—N5—C30	−177.3 (2)	Cl2—C16—C17—C18	−179.79 (18)
C6—C1—C2—C3	1.9 (3)	C16—C17—C18—C19	−2.0 (4)
Cl1—C1—C2—C3	−177.79 (15)	C17—C18—C19—C20	2.3 (3)
C1—C2—C3—C4	0.0 (3)	C17—C18—C19—C22	−176.9 (2)
C2—C3—C4—C5	−1.7 (3)	C18—C19—C20—C21	−1.0 (3)
C2—C3—C4—C7	174.90 (17)	C22—C19—C20—C21	178.2 (2)
C3—C4—C5—C6	1.6 (3)	C19—C20—C21—C16	−0.5 (4)
C7—C4—C5—C6	−174.93 (17)	C17—C16—C21—C20	0.8 (4)
C4—C5—C6—C1	0.2 (3)	Cl2—C16—C21—C20	−179.00 (18)
C2—C1—C6—C5	−2.0 (3)	C18—C19—C22—O2	−9.3 (3)
Cl1—C1—C6—C5	177.71 (14)	C20—C19—C22—O2	171.49 (17)
C3—C4—C7—O1	−17.5 (2)	C18—C19—C22—C28	−125.2 (2)
C5—C4—C7—O1	158.99 (16)	C20—C19—C22—C28	55.6 (2)
C3—C4—C7—C13	−135.12 (17)	C18—C19—C22—C23	114.7 (2)
C5—C4—C7—C13	41.3 (2)	C20—C19—C22—C23	−64.5 (2)
C3—C4—C7—C8	100.45 (19)	O2—C22—C23—C24	65.7 (2)
C5—C4—C7—C8	−83.1 (2)	C19—C22—C23—C24	−59.0 (2)
O1—C7—C8—C10	−179.60 (15)	C28—C22—C23—C24	179.8 (2)
C4—C7—C8—C10	59.5 (2)	O2—C22—C23—C25	−59.9 (2)
C13—C7—C8—C10	−61.4 (2)	C19—C22—C23—C25	175.41 (19)
O1—C7—C8—C9	55.3 (2)	C28—C22—C23—C25	54.2 (2)
C4—C7—C8—C9	−65.6 (2)	C24—C23—C25—C26	142.2 (2)
C13—C7—C8—C9	173.47 (16)	C22—C23—C25—C26	−91.9 (3)
C9—C8—C10—C11	−146.64 (19)	C24—C23—C25—C27	72.4 (3)
C7—C8—C10—C11	87.6 (2)	C22—C23—C25—C27	−161.6 (3)
C9—C8—C10—C12	−76.2 (2)	C23—C25—C26—C27	−106.9 (3)
C7—C8—C10—C12	157.97 (18)	C23—C25—C27—C26	111.7 (3)
C8—C10—C11—C12	106.7 (2)	C29—N4—C28—C22	57.1 (3)
C8—C10—C12—C11	−112.3 (2)	N5—N4—C28—C22	−124.8 (2)
C14—N1—C13—C7	−82.3 (2)	O2—C22—C28—N4	−71.1 (2)
N2—N1—C13—C7	105.16 (18)	C19—C22—C28—N4	49.6 (2)
O1—C7—C13—N1	45.42 (19)	C23—C22—C28—N4	170.39 (16)
C4—C7—C13—N1	164.46 (14)	N5—N4—C29—N6	−1.7 (3)
C8—C7—C13—N1	−70.87 (19)	C28—N4—C29—N6	176.6 (2)
C15—N3—C14—N1	0.2 (2)	C30—N6—C29—N4	1.4 (3)
N2—N1—C14—N3	−0.5 (2)	N4—N5—C30—N6	−0.4 (3)
C13—N1—C14—N3	−173.81 (16)	C29—N6—C30—N5	−0.6 (3)

### Hydrogen-bond geometry (Å, º)

*Cg*1 is the centroid of the N1/N2/C15/N3/C14 ring.

**Table:**

*D*—H···*A*	*D*—H	H···*A*	*D*···*A*	*D*—H···*A*
O1—H1···N6^i^	0.84	2.05	2.884 (2)	171
O2—H2···N3^ii^	0.84	2.07	2.856 (2)	156
C8—H8···Cl1^iii^	1.00	2.83	3.633 (2)	137
C15—H15···O2^iv^	0.95	2.53	3.284 (2)	137
C13—H13*B*···*Cg*1^v^	1.00	2.91	3.473 (2)	117

Symmetry codes: (i) *x*−1, *y*, *z*−1; (ii) *x*, *y*, *z*+1; (iii) *x*+1, *y*, *z*; (iv) −*x*+2, −*y*+1, −*z*+1; (v) −*x*+1, −*y*+1, −*z*.

## References

[bb1] Brandenburg, K. (2010). *DIAMOND*. Crystal Impact GbR, Bonn, Germany.

[bb2] Bruker (2013). *APEX2*, *SAINT* and *SADABS*. Bruker AXS Inc., Madison, Wisconsin, USA.

[bb3] Chopra, D., Mohan, T. P., Rao, K. S. & Guru Row, T. N. (2004). *Acta Cryst.* E**60**, o2410–o2412.

[bb4] Hester, S., Moore, T., Padgett, W. T., Murphy, L., Wood, C. E. & Nesnow, S. (2012). *Toxicol. Sci.* **127**, 54–65. 10.1093/toxsci/kfs086 22334560

[bb5] Sheldrick, G. M. (2008). *Acta Cryst.* A**64**, 112–122. 10.1107/S0108767307043930 18156677

[bb6] Sheldrick, G. M. (2015). *Acta Cryst.* C**71**, 3–8.

