# Supplementary figures and images for: Crystal structure of cyproconazole
Source: Acta Crystallogr E Crystallogr Commun. 2015 Nov 28;71(Pt 12):o1007. doi: 10.1107/S2056989015022665 (PMC4719948; doi:10.1107/S2056989015022665)

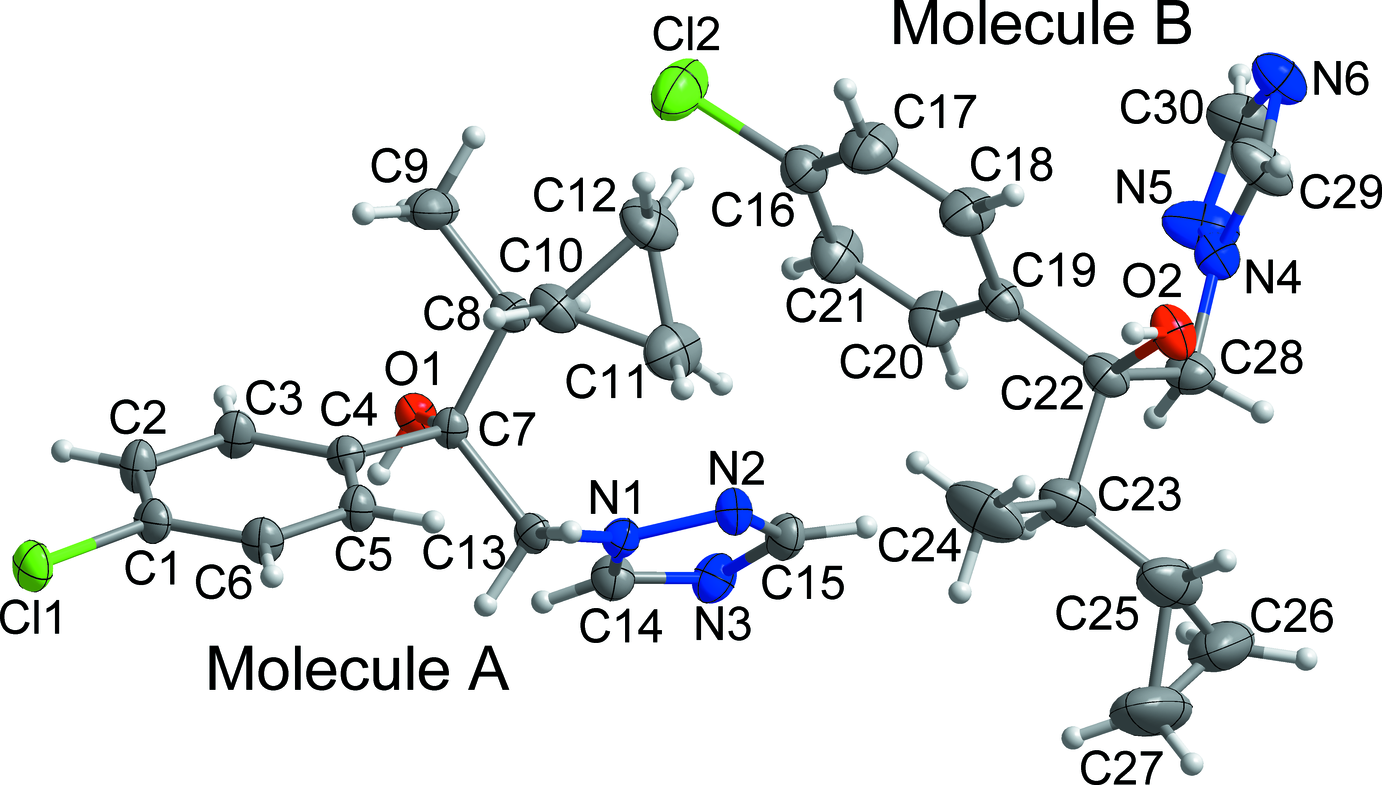

Supplement: Supplementary file 4 [file e-71-o1007-fig1.tif]

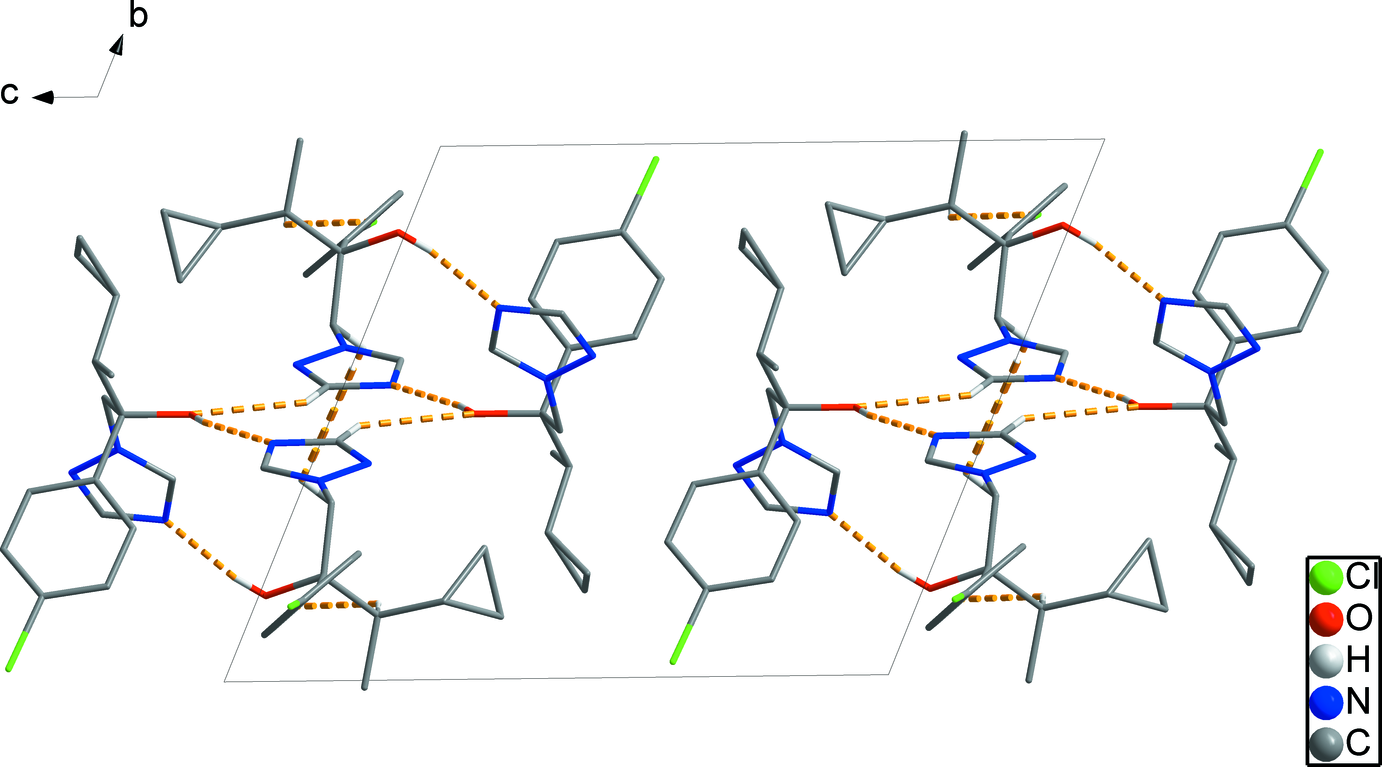

Supplement: Supplementary file 5 [file e-71-o1007-fig2.tif]
